# Beneficial effect of compound essential oil inhalation on central fatigue

**DOI:** 10.1186/s12906-018-2375-6

**Published:** 2018-11-26

**Authors:** Chenxia Han, Feng Li, Simin Tian, Yan Liu, Huai Xiao, Xiumei Wu, Weiyue Zhang, Wei Zhang, Meng Mao

**Affiliations:** 1 Basic Medicine School, Beijing University of Chinese Medicine, Beijing, People’s Republic of China; 2 Chinese Medicine School, Beijing University of Chinese Medicine, Beijing, People’s Republic of China; 3 Insect Biological Medicine Research Institution, Dali University, Dali, Yunnan People’s Republic of China

**Keywords:** Essential oil, Central fatigue, Traditional Chinese medicine

## Abstract

**Backgrounds:**

Although the physical and mental enhancement effect of essential oils have been proved, the beneficial effect of essential oil in central fatigue remains unclear. In this study, we extracted essential oils from nine aromatic plants to make a compound essential oil, and detected the therapeutic effect of central fatigue by daily aerial diffusion.

**Methods:**

Thirty-three rats were randomly and equally divided into control group, chronic sleep deprivation group, and compound essential oil inhalation group. Central fatigue was generated by chronic sleep deprivation.

**Results:**

After 21-day various interferences, it is found that the sleep deprivation rats showed an evident decrease in physical endurance, negative emotion, and cognitive dysfunction compared with the control group, and the group that treated with the compound essential oil behaved significantly better than central fatigue group.

**Conclusion:**

We concluded that this formula of essential oils could alleviate central fatigue on rats, and our study provides a new direction of application of aromatic therapy, which could be expanded to insomnia, depression and other healthy issue in the further research.

## Introduction

A lot of clinical evidence has proved that aromatherapy could improve the brain function and alleviate fatigue [1]. The term ‘aromatherapy’ was coined by French chemist and perfumiér René Maurice Gattefossé in the 1920s [2]. Aromatherapy has been used as an effective method in complementary and alternative medicine, owning to its noninvasive operation, convenience in administration and fast results. Essential oils have been widely used as a treatment in body relaxing, mood enhancement, pain relief, anti-stress, improving cognitive efficiency, spirit well-being and many other psychological and physical conditions [3]. It can directly act on respiration system [4], circulation system [5], and central nervous system via skin and respiration tract. The basic mechanism of aromatherapy is the inhalation of volatile ingredients into respiration system, or absorption of monomer ingredients into skin, finally take effect in certain organ. Some research argue that the connection between olfaction and the limbic system in the brain may be the main mechanism of aromatic therapy [1]. The scent receptors in the nose could send chemical messages via the olfactory nerve to the brain’s limbic region, and then regulate the body function including blood pressure, breath, and emotion [6]. However, opinions based on this theory are controversial. Another research proposed that the augmentation of GABA could be the basic mechanism of lavender essential oil ameliorating convulsions in mice [7]. Studies focused on the molecular mechanism during aromatic functioning with significance remains absent.

Central fatigue was first reported in 1904 by A. Mosso [8]. It represents a failure to complete mental and physical tasks in the absence of demonstrable cognitive failure or motor weakness [9], and could be resulted from the dysfunction of central nerves system [10]. Clinical manifestation related to central fatigue reported in current literatures mainly including fatigue sensation, weakness of physical function, low efficiency during mental task, and negative emotions including anxiety, depression [11]. It is a complex state which could be induced by massive physical and/or mental tasks [12]. Several neurotransmitters such as 5-HT and DA have been studied to be related to central fatigue [13, 14]. Although relax therapy [15] and sufficient oxygen [16] could improve the situation, an effective and suitable medical intervention for central fatigue remains absent.

The aromatherapy plays an important role in fatigue treatments due to the positive physical and mental effects [17]. It is known that relax therapy could alleviate central fatigue cause it could relax the intensive state of central nervous system and muscle [15]. And it could improve sleep and depression, and the whole well-being in cancer patients [18]. As an important role in relaxing treating, essential oils inhalation has been proved to be effective in mental exhaustion and burnout [19], and the prevention and treatment of stress and fatigue as well [20]. In addition, accumulated evidence of clinical trials has indicated that the intractable fatigue and other conditions caused by cancer [21, 22] and radiotherapy [23, 24], could be relieved by essential oil. Although some clinical evidence indicated the effectiveness on burn out and mental fatigue, few research has investigated the effect of this treatment on central fatigue.

Sleep deprivation could generate central fatigue on rats [25]. As we introduced above, essential oil inhalation could improve fatigue, and it could improve sleep issues as well [26], due to its central function enhancement and spirit well-being effects. Thus, in this research, we employed chronic sleep deprivation to generate central fatigue on rats [27], and used a compound essential oil made from selected natural plants, which have been proved to exhibited evident effect on mental disorders [28, 29], cognitive tasks [30], negative emotions [31]. We reported a pharmacodynamics study of compound essential oil in central fatigue, by analyzing central fatigue related measurement such as physical endurance, emotion, decision-making capacity, locomotor activity through behavioral tests, to evaluate the beneficial properties of this compound essential oil.

## Materials and methods

### Animals

Male (weighing 200 ± 10 g) Wistar rats were purchased from Beijing Vital River Laboratory Animal Technology Limited Company (Beijing, China). The animals were maintained in a room with a constant temperature of 23 ± 1 °C; a relative humidity of 30–40%; light for 12 h from 06:00 to 18:00; and ad libitum food and purified water. Thirty three rats were randomly divided into three groups as follows: control group (CON, rats were fed routinely for 21 days, *n* = 11), chronic sleep deprivation group (CSD, rats were promoted by chronic sleep deprived for 21 days, *n* = 11), compound essential oil group (CEO, rats were promoted with chronic sleep deprived for 21 days with compound essential oil inhalation every day, *n* = 11).

The rats were anaesthetised with an intraperitoneal injection of 10% pentobarbital sodium (4 ml/kg) and subsequently sacrificed by rapid decapitation.

The experiments were approved by the Institutional Animal Ethics Committee of Beijing University of Chinese Medicine. All animals were maintained in accordance with the guidelines outlined by the Chinese legislation on the ethical use and care of laboratory animals. All efforts were made to minimize both animal suffering and the number of animals used to produce reliable data.

### Compound essential oil preparation and administration

The compound essential oil was a mixture formula including nine natural plants essential oils. It consist of *Santalum album*, *Citrus aurantium*, *Citrus limonum*, *Styrax benzoin*, *Citrus paradisi*, *Mentha piperata*, *Acori tatarinowii rhizoma*, *Rhodiolae crenulatae radix et rhizoma*, and *Camellia sinensis (linn.)o. ktze*.

Pure essential oils of *Santalum album*, *Citrus aurantium*, *Citrus limonum*, *Styrax benzoin*, *Citrus paradisi, Mentha piperata*, were purchased from Beijing Piaowang Science Technology Co, Ltd.(China). Essential oils of *Acori tatarinowii rhizoma*, *Rhodiolae crenulatae radix et rhizoma,* and *Camellia sinensis (linn.)o. ktze* were prepared follow the method in Pharmacopoeia of People’s Republic of China [32] respectively. In detail, the crude drug were purchased from Beijing Tongrentang Drug Strore (China), drug(100 g per drug) was boiled in 1000 ml distilled water for 6-7 h in a heating laboratory flask, the essential oil was collected using a condenser pipe during the boiling. Finally, the three collected essential oil of *Acori tatarinowii rhizoma, Rhodiolae crenulatae radix et rhizoma, and Camellia sinensis (linn.)o. ktze* was mixed with purchased single essential oils, together to make a compound essential oil with a specific mixing proportion. (The mixing ratio of *Santalum album*, *Citrus aurantium*, *Citrus limonum*, *Styrax benzoin*, *Citrus paradisi, Mentha piperata*, *Acori tatarinowii rhizoma, Rhodiolae crenulatae radix et rhizoma,* and *Camellia sinensis (linn.)o. ktze* was 8:4:4:5:1:6:0.2:0.2:0.2).

Rats in CEO group were taken to a separate room whose environment was same as the original experiment room for inhalation of compound essential oil for 45 min every training day on 9:00 am. Essential oils was inhaled by adding 100 μl to 300 ml water in an aroma humidifier which spread the aroma throughout the room. After inhalation, they were taken back to the experiment room [33].

### Gas chromatography–mass spectrometry analysis

GC-MS was performed with gas chromatography instrument (Agilent Technologies 7890A) coupled to a mass spectrometer (Agilent Technologies 5975C). Compounds were separated on aHP-5 MS capillary column (Agilent, 30 m × 0.25 mm, 0.25 μm).

The column temperature was maintained for 1 min at 40 °C during desorption, then ramped to 280 °C at 5 °C/min, and kept for 3 min at 280 °C. Splitless injection was conducted and helium was used as carrier gas with the flow-rate of 1.0 mL/min. The spectrometer was operated in electron-impact (EI) mode with the scan range 50–550 m/z, the ionization energy 70 eV, and the scan rate 0.2 s per scan. The ionization source temperature was 230 °C.

The volatile components were identified by mass spectral comparison with the spectra of reference compounds in National Institute of Standards and Technology (NIST) mass spectral library.

### Chronic sleep deprivation

The chronic sleep deprivation was generated by modified multiple platform method [34]. This method has been reported to interfere with total sleep, mainly rapid eye moved sleep [35]. Rats in CSD group were deprived sleeping for 14 h per day from 18:00 to 8:00, last for 21 days, according to our previous research [36]. The equipment for modified multiple platform method was made with plastic, and there are 15 platforms placed on the bottom of the tank (110 × 60 × 40 cm); they were surrounded by water at a temperature of 20–22 °C at a depth of 1.0 cm below the platform surface; iron cages and bottles were filled with food and water, respectively, on the top of the tank. Thus, the rats could move around inside the tank by jumping from one platform to another. When rats fall asleep, they will fall into the water and wake up.

### Weight-loaded forced swimming (WFS)

WFT was promoted after 21-day training followed the described method [37]. Rats were forced to swim individually in a plastic pool which was filled with water at a temperature of 20–22 °C and a depth of 60 cm. A tin wire (10% of body weight) was loaded on the tail root of each rat. The endurance capacity was recorded as the time rat began to swim till exhausted. The rats were assessed to be exhausted when they failed to rise to the surface of water to breathe within a10 s period. At swimming session, rats were taken out from the water and dried with a towel, and put back in their home cages. Water was drained after each rat swimming.

### Open field test (OFT)

OFT was promoted after 21-day training. This test provides a novel environment in which to measure animal locomotion, exploration, and anxiety [38]. The open field arena (100 × 100 × 40 cm) is constructed of acrylic, with gray walls and a black floor, which is divided into 25 equally sized areas, as previously described [39]. The time spent in central area, number of crossing squares, total distance travelled, maximum continuous distance travelled, the mean velocity, time and frequency of vertical activity, time and frequency of grooming behavior and the number of defecations were measured. Each rat was tested for 5 min. Measures were assessed using Etho Vision XT software (Nodule, the Netherlands). Each rat was only tested once. The arena was thoroughly cleaned with 75% ethanol between rats during test.

### Elevated plus maze (EMP)

EMP is an effective tool to evaluate the anxiety on rodents [40]. The elevated plus maze was constructed in the end of training day as previously described [41], with two open arms (30 × 5 × 15 cm) and two closed arms (30 × 5 × 15 cm) that extended from a central, open square (5 × 5 cm). The maze was elevated on a pedestal to a height of 45 cm above the floor. Three measures were tested: the amount of time to explore the open arms relative to the total amount of time to explore the open and closed arms of the maze, recorded as the ratio of time spent in the open arms/time in the arms; and the total number of entries into the open arms relative to the total entries, recorded as the ratio of number of open arms entries/total entries; the time spent in central area. Each rat was tested for 3 min. Measures were assessed using EthoVision XT software (Nodule, the Netherlands). Rat was tested individually once. The maze was thoroughly cleaned with 75% ethanol between rats during test.

### Statistical analysis

The data are expressed as the mean ± standard error of the mean. All data were initially tested for normality and homogeneity of variance and then analyzed using one-way analysis of variance (ANOVA) or a Kruskal-Wallis test. In addition, the least significant difference (LSD) or Mann-Whitney test was adopted for group comparisons. The statistics of ANOVA and Kruskal-Wallis tests were represented as F and H respectively. All data were analyzed with Statistical Package for the Social Sciences (SPSS) software, version 17.0 (SPSS, Chicago, IL, USA). *P* values < 0.05 were considered statistically significant.

## Results

### GC-MS analysis

**Fig. 1:**
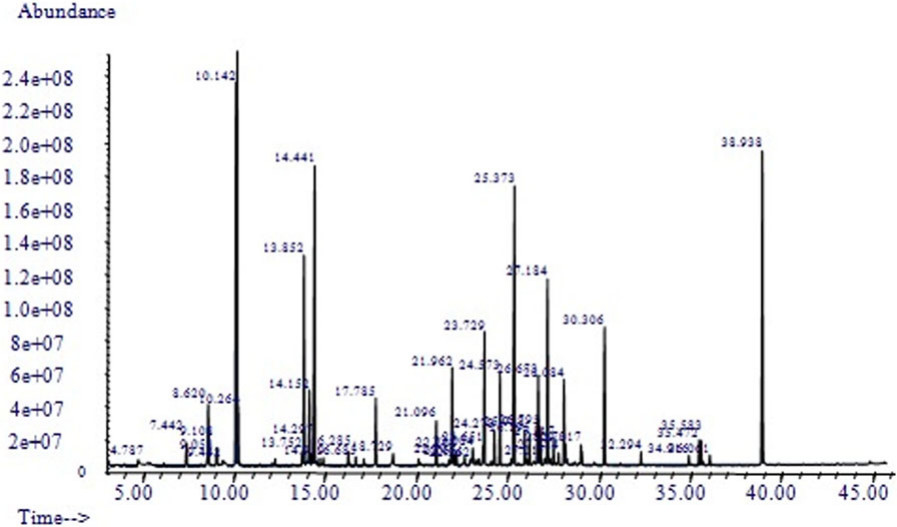
Peak marked with retention time. The result of GC-MS analysis of the compound essential oil, the main components were showed as the marked peak with retention time

The approximate relative amounts of individual components were expressed as peak area relative to the total peak area. The peak marked with retention time was shown in Fig. 1, and the components were shown in Table 1.

**Table 1:** Identified components of the compound essential oil

No.	Retention time (min)	Relative Content (%)	Compound	Molecular weight	Similarity (%)
1	4.787	0.55	hexamethyl cyclotrisiloxane	222.056	95
2	7.442	0.86	(1S)-2,6,6-trimethylbicyclo[3.1.1]hept-2-ene	136.125	97
3	8.62	2.31	(1S)-6,6-dimethyl-2-methylene-bicyclo[3.1.1]heptane	136.125	97
4	9.053	0.30	(−)		
5	9.108	0.38	(−)		
6	9.442	0.27	(−)		
7	10.142	15.46	Tricyclo[5.3.0.0(3,9)]decane	136.125	90
8	10.264	0.78	eucalyptol	154.136	98
9	13.752	0.40	trans-5-methyl-2-(1-methylethyl)- cyclohexanone	154.136	98
10	13.852	5.72	cis-5-methyl-2-(1-methylethyl)-cyclohexanone	154.136	93
11	14.152	3.04	l-menthone	154.136	96
12	14.297	0.39	(1α,2β,5α)-5-methyl-2-(1-methylethyl)-cyclohexanol	156.151	91
13	14.441	10.31	2,6-dimethyl-2,6-octadiene	138.141	90
14	14.919	0.27	alpha-terpineol	154.136	93
15	16.285	0.47	pulegone	152.12	98
16	16.685	0.27	3-methyl-6-(1-methylethyl)-2-cyclohexen-1-one	152.12	95
17	17.785	1.67	menthyl acetate	198.162	91
18	18.729	0.48	dodecamethyl-cyclohexasiloxane	444.113	90
19	21.096	1.22	caryophyllene	204.188	99
20	21.962	3.25	(−)		
21	22.107	0.32	(1S-exo)-2-methyl-3-methylene-2-(4-methyl-3-pentenyl)- bicyclo[2.2.1]heptane	204.188	90
22	22.207	0.38	ethyl- 3-phenyl-2-propenoic acid ester	176.084	98
23	22.651	0.25	1-(1,5-dimethyl-4-hexenyl)-4-methyl-benzene	202.172	98
24	22.962	0.25	di-epi-α-cedrene	204.188	96
25	23.084	0.66	tetradecamethyl-cycloheptasiloxane	518.132	91
26	23.651	0.51	(1S-cis)-1,2,3,5,6,8a-hexahydro-4,7-dimethyl-1-(1-methylethyl)-naphthalene,	204.188	97
27	23.729	3.63	(−)		
28	24.273	0.85	[1R-(1α,3α,4β)]-4-ethenyl-4-trimethyl-3-(1-methylethenyl)-cyclohexanemethanol	222.198	91
29	24.573	2.32	nerolidol	222.198	91
30	25.373	10.19	(−)		
31	25.928	0.98	8-epi-γ-eudesmol	222.198	95
32	26.195	0.84	gama.-eudesmol	222.198	99
33	26.673	3.66	(−)		
34	26.795	0.91	7-epi-α-selinene	204.188	95
35	27.017	0.37	(−)		
36	27.184	5.14	(−)		
37	27.517	0.69	Z-α-trans-bergamotol,	220.183	91
38	27.784	0.33	E-cis,epi-α-santalol,	220.183	97
39	28.084	2.38	(−)		
40	28.173	0.52	methyl tetradecanoate	242.225	99
41	29.017	0.89	Cis-lanceol	220.183	90
42	30.306	3.66	isopropyl myristate	270.256	95
43	32.294	0.33	methyl hexadecanoic acid ester	270.256	99
44	34.916	0.26	13-hexyloxacyclotridec-10-en-2-one	280.24	93
45	35.472	0.64	(E,E)- methyl-9,12-octadecadienoic acid ester	294.256	99
46	35.583	0.66	Methyl-11-octadecenoic acid ester	296.272	99
47	36.061	0.27	methyl stearate	298.287	99
48	38.938	9.70	[R-(Z)]-12-hydroxy-, methyl-9-octadecenoic acid ester	312.266	94

(−) not identified

According to the analysis of GC-MS, there are 48 components in the compound essential oil, 38 of them has been identified. The highest relative content is *Tricyclo[5.3.0.0(3,9)] decane*, with a proportion of 15.46%, followed by *2,6-dimethyl-2,6-octadiene*(10.31%) and *[R-(Z)]-12-hydroxy-, methyl-9-octadecenoic acid ester*(9.7%).

### Bodyweight

**Fig. 2:**
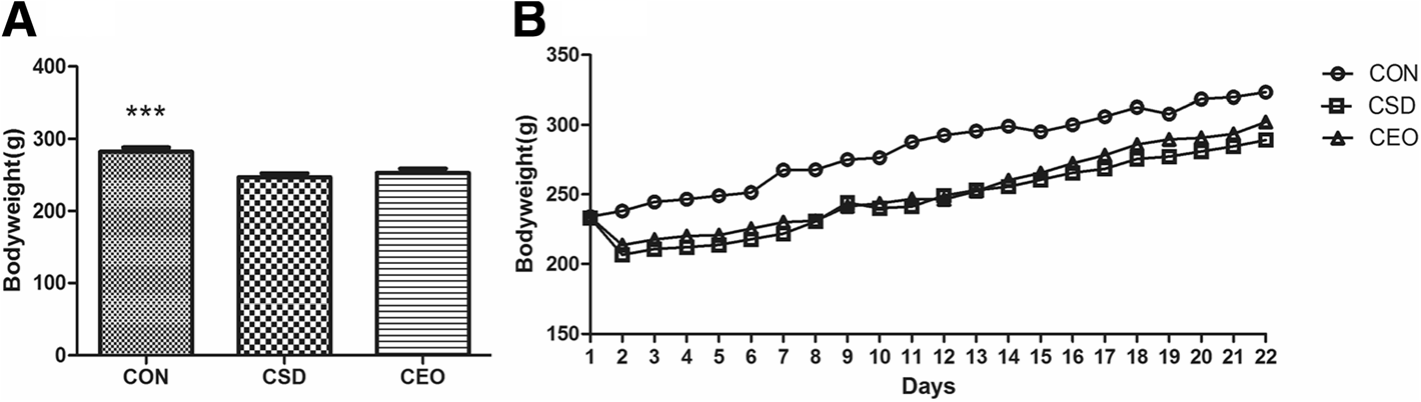
Bodyweight. All data were represented as the mean ± SEM (*n* = 11), ***refers to *p* < 0.001 vs CSD group. The bodyweight of CON rats increased significantly compared with the CSD rats (**a**). Details of daily body weight changes, as recorded at 9:00 during training days (**b**). The growth of bodyweight of CON group exhibited a higher growing trend

There was a significant difference among the three groups in daily bodyweight [F(2,63) = 10.304, *p* = 0.000]. The bodyweight of CSD group showed a significant decrease compared with CON group (*p* = 0.000). However, there was no significant difference seen between CSD and CEO group in the LSD test (*p* = 0.482). Similarly, the growing trend of CON group was obviously higher than the other two groups during the experimental days (Fig. 2).

### Access of WST

**Fig. 3:**
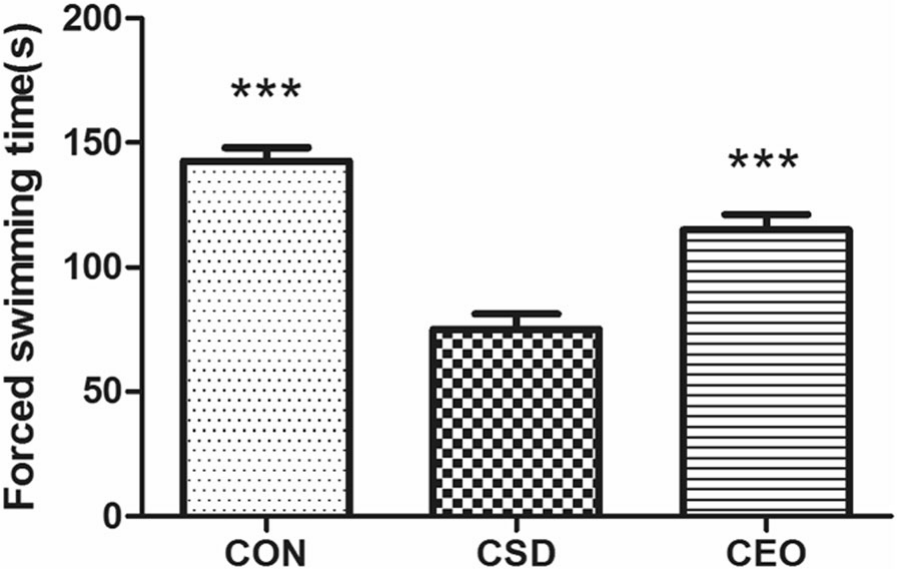
Swimming time of weight loaded forced swimming test. All data were represented as the mean ± SEM (*n* = 11), *** refers to *p* < 0.001 vs CSD group. The swimming time of CON group was significantly longer compared with CSD group, CEO increased the swimming time significantly compared with the CSD group

The result of WST was significantly different [F (2,30)=33.738, *P* = 0.000]. CSD evidently cut down the swimming endurance time compared with CON group (*p* = 0.000), and CEO extended the swimming time significantly (*p* = 0.000) in LSD test (Fig. 3).

### Access of OFT

**Fig. 4:**
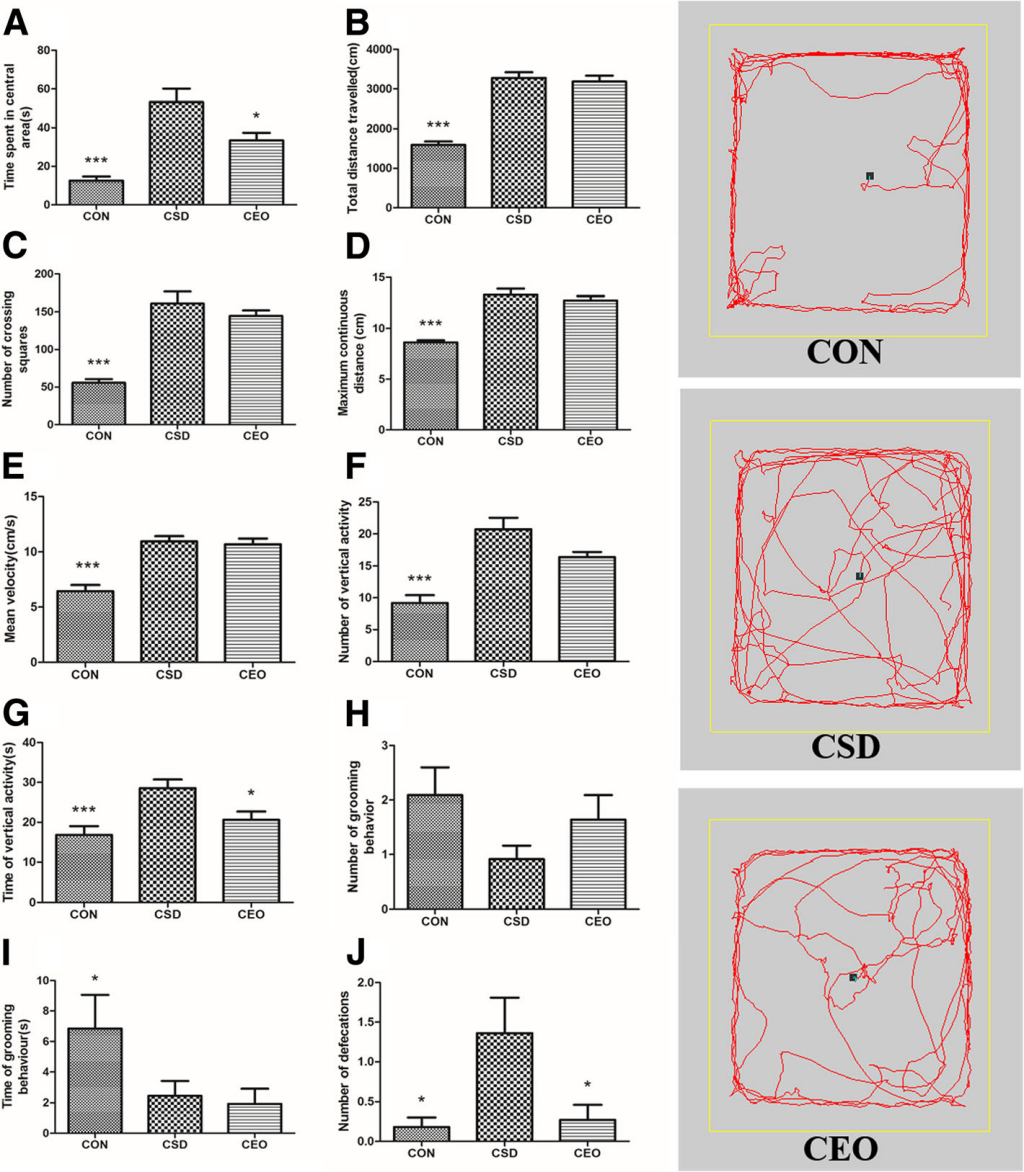
Assessment of open field test. All indexes were recorded in a test of 5 min. All data were represented as the mean ± SEM (*n* = 11), * refers to *p* < 0.05, *** refers to *p* < 0.000 vs. CSD group. The time spent in center of CON group was significantly shorter compared with CSD group, and CEO decreased the central time (**a**). The total distance travelled (**b**), number of crossing squares (**c**), max continuous distance (**d**), mean velocity (**e**) of CON group all decreased significantly compared with CSD group respectively, and treatment of CEO showed a reduction trend on these parameters. Number and time recorded of vertical activity (**f**, **g**) showed the same situation as horizontal activity. No significant difference was found in the number of grooming behavior (**h**). The time of grooming (**i**) in CON group were significantly higher than CSD group. Number of defecations (**j**) of CSD group increased significantly compared with CON group, and CEO was seen to decrease the measure significantly

The analysis of parameters in OFT was showed in Fig. 4. The time spent in central area result in significant difference among the groups in Kruskal-Wallis test [H(2)=19.531, *p* = 0.000]; in the Mann-Whitney test, the central time in CSD group exhibited a evident increase compared with the CON group (*p* = 0.000), and CEO decreased the one compared with CSD group(*p* = 0.028). The parameters of horizontal activity showed a consistent result that the CSD rats enhanced these parameters significantly while CEO decreased them without significant difference, including total distance travelled [H(2)=21.392, *p* = 0.000; CON vs CSD: *p* = 0.000; CSD vs CEO: *P* = 0.768], number of crossing squares [H(2)=21.666, *p* = 0.000; CON vs CSD: *p* = 0.000; CSD vs CEO: *P* = 0.412], maximum continuous distance travelled [H(2)=21.383, *p* = 0.000; CON vs CSD: *p* = 0.000; CSD vs CEO: *P* = 0.869], and mean velocity [F(2,30)=23.755, *p* = 0.000; CON vs CSD: *p* = 0.000; CSD vs CEO: *P* = 0.726]. On the other hand, vertical activity was detected as number [H(2)=21.608, *p* = 0.000; CON vs CSD: *p* = 0.000; CSD vs CEO: *P* = 0.054] and time [H(2)=12.731, *p* = 0.002; CON vs CSD: *p* = 0.001; CSD vs CEO: *P* = 0.017] of standing on hind feet of rats, the CSD enhanced both number and time of vertical activity, while CEO reduced them. There was no significant difference in the number of grooming behavior [H(2)=5.864, *p* = 0.053], and the time of grooming resulted in significant difference [H(2)=9.729, *p* = 0.008; CON vs CSD: *p* = 0.027; CSD vs CEO: *P* = 0.716], no significant difference was found between CSD and CEO groups in grooming behavior. Number the defecations resulted in significant difference [H(2)=7.669, *p* = 0.022], the defecations of CSD group increased significantly compared with the CON group (*p* = 0.019), and CEO decreased the number compared the CSD group (*p* = 0.032).

### Access of EPM

**Fig. 5:**
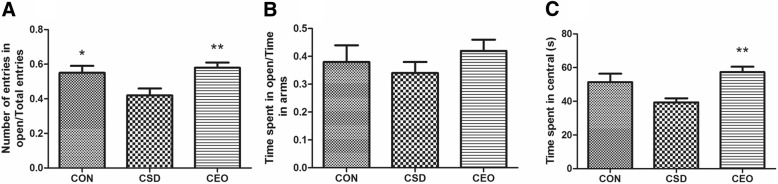
Assessment of elevated plus maze. All data were represented as the mean ± SEM (*n* = 11), * refers to *p* < 0.05 **refers to *p* < 0.01 vs. CSD group. The ratio of number of entries in the open /total entries (**a**) in CSD group decreased significantly compared with CON group. CEO increased the ratio. There was no significant difference in the ratio of time spent in open/total arms (**b**), whereas the trend showed same as before. The reduction of central time (**c**) in CSD was not significant, however the treatment CEO increased the measure compared with CSD group

The ratio of entries in the open arms changed significantly among the three groups [F(2,30)=5.728, *P* = 0.008]. Compared the CSD group, the rats in CON (*p* = 0.012) and CEO (*p* = 0.004) groups visibly visited the open arms more frequently. However, the other measure that explain the open arm visiting which recorded as the ratio of time in open arms resulted in no significant difference changes [H(2)=1.589, *p* = 0.045], also the trend of changing was similar to the former measure. Time spent in the central area, which reflects the decision making ability, showed a significant difference [H(2)=9.419, *p* = 0.009]. Although the central time in CSD group decreased without significance (*p* = 0.224), the index of CEO group increased significantly compared with the CSD group (*p* = 0.001) (Fig. 5).

## Discussion

Aerial diffusion is a fast way for aromatic therapy to bring into effect since it takes only 4 s to induce the response of central nervous system [42]. It takes advantage of respiration administration which begins with the absorption of volatile molecules through the nasal mucosa [43], meanwhile, the volatile molecules get into the lung followed by gas exchanging, then reach circulation system. Odor molecules are transformed into chemical signals, which could stimulate the releasing of neurotransmitters in brain, then balance the nerve system [44]. This mode of operation can effectively influence the functional brain changes, especially mental disorder and mood. Central fatigue is a common condition revolving cognitive dysfunction, negative emotion, and physical weakness. Our study indicated that the inhalation of compound essential oil can directly attenuate negative behavioral changes after central fatigue on rats.

It has been proved that *Citrus limonum* [45], *Citrus paradise* [46], *Citrus aurantium* [47], *Mentha piperata* [48]*, Santalum album* [49] have the beneficial effects on stress, anxiety, depression, oxidant [50], oxidant stress, and also could modulate sympathetic activity. Meanwhile, *Styrax benzoin* [51], *Rhodiolae crenulatae radix et rhizoma* [52]*, Camellia sinensis (linn.)o. ktze* [53] and *Acori tatarinowii rhizoma* [54] all have been studied to anti-fatigue effectively by regulating energy process, reducing metabolism products, improve the function of Brain Blood Barrier. Since no evidence has been found that the compounds of these gradients could ameliorating central fatigue, this study proved that the compound essential oil inhalation was effective.

According to the result of GC-MS analysis, there are 5 main groups of material, esters, alcohols, terpenes, alkanes, and alkenes. The eaters and alcohols could inspire the central nervous system [33], while the terpenes provide the anti-oxidative effect [55]. Among these identified components, *Tricyclo[5.3.0.0(3,9)] decane* is the highest relative content component in the compound essential oil, in addition, *2,6-dimethyl-2,6-octadiene*, and *[R-(Z)]-12-hydroxy-, methyl-9-octadecenoic acid ester* also have much higher proportion than other identified components. However, the compound essential oil alleviated central fatigue as a whole formula, it is difficult to conclude that the highest contents are the active ingredients in the compound essential oil. The deeper mechanism of these chemical substance on central fatigue remains further study.

The CEO group showed increased physical function. It is studied that central fatigue has a strong negative impact on the physical performance [16]. The WFS test reflects the entire fatigue state especially physical endurance by the swimming time. The swimming time of CSD rats decreased evidently, which exhibited a weakness of body function. Compared with the non-treated CSD rats, rats in CEO group showed a longer endurance during the WFS test, suggested that the inhalation of compound essential oil could improve the physical function. The body weight could be affected by two reasons: the food intake and the energy consumption. Since central fatigue is closely related to emotion, the appetite is supposed to be affected and result in lower food intake. Bodyweight can reflect the motivation in food intake, and the energy consumption and metabolism as well during central fatigue. However, the bodyweight was showed on significant change after inhalation treatment.

The compound essential oil improved behavioral performance associated with anxiety and cognitive function. The Open field test is widely used in emotion evaluation on rodents, especially, anxiety. Thigmotaxis (preference for peripheral over central parts of the open field) was recorded as time spent in the central arena [56]. Evidently increasing central time reflects a lower capacity in space cognition because normal rats would realize the environment then rapidly leave from the novel and open filed center space [57]. Given that the CSD increased central time, there was an evident decrease after compound essential oil treatment that indicating the enhancement of space cognize capacity. Consist with our previous research [37], the increasing central time represented a high-stress state and anxiolytic-like behavior of rodent, which has also been relieved by CEO. Locomotor activity was composed by horizontal activity and vertical activity. It has been reported that physical fatigue could decrease locomotor activity [58], unfortunately, few literature reported the changes in locomotor activity during central fatigue. The measures of horizontal activity in this study composing total distance travelled, number of crossing squares, maximum distance travelled, and the mean velocity, it is showed a significant increase in CSD rats, and a decreasing trend after inhalation treatment without statistical significance. We hypothesized this result can reflect a high-stress state in CSD rats, accompanying by fear and irritability, and the relaxing property of compound essential oil attenuated these behavioral changings. Similarly, the two parameters of vertical activity in CSD, namely the number and total time of vertical activity, exhibited an evident enhancement, which were decreased in CEO group, implying the antianxiety action of inhalation treatment. Grooming behavior and defecation also reflect anxiety, in this study, a reduction of grooming was seen in CSD group, and CEO improved the grooming occurrence without significance. Although there was a slight, non-significant decrease in CEO group of grooming time, we consider this could be ignored. An evident increase of defecation number was observed in CSD group during the test, meanwhile an obvious decrease was showed in CEO group, both suggesting an improvement of essential oil in stress, fear and anxiety.

Elevated plus maze is a classic paradigm in anxiety evaluation. The ratio of entries number in open of CSD group went through a decrease, and CEO rats visited the open arms more frequently. The same trend was seen in the other measure, ratio of time in open arm, but without significance. Thus it could demonstrate a property of compound essential oil in anti-anxiety during central fatigue. In addition, this test not only assessed factors related to anxiety, but also evaluates the subjects’ risk assessment and decision-making ability by measuring the time spent in the center. It has been found that less time spent in central represents a weakness of this ability [59]. Although the central time of CSD group decreased without significance, there was an evident increase in CEO group, which implies a clue in effect of high level brain cognitive function in our essential formula.

## Conclusion

The compound essential oil could attenuate central fatigue on rats by enhancing physical endurance, reducing negative emotion as decreasing depression and anxiety-like behavior, and improving space cognition and decision-making ability.

## Abbreviations

CEO: Compound essential oil group
CON: Control group
CSD: Chronic sleep deprivation group
EPM: Elevated plus maze
OFT: Open field test
WFS: Weight-loaded forced swimming
